# A computational study of global optimization solvers on two trust region subproblems

**DOI:** 10.1007/s10898-018-0649-7

**Published:** 2018-04-12

**Authors:** Tiago Montanher, Arnold Neumaier, Ferenc Domes

**Affiliations:** Faculty of Mathematics, University of Vienna, Oskar-Morgenstern-Platz 1, 1090 Vienna, Austria

**Keywords:** Reliability analysis, Cluster effect, Branch-and-bound solvers, SDP-relaxations, Celis–Dennis–Tapia subproblem

## Abstract

One of the relevant research topics to which Chris Floudas contributed was quadratically constrained quadratic programming (QCQP). This paper considers one of the simplest hard cases of QCQP, the two trust region subproblem (TTRS). In this case, one needs to minimize a quadratic function constrained by the intersection of two ellipsoids. The Lagrangian dual of the TTRS is a semidefinite program (SDP) and this result has been extensively used to solve the problem efficiently. We focus on numerical aspects of branch-and-bound solvers with three goals in mind. We provide (i) a detailed analysis of the ability of state-of-the-art solvers to complete the global search for a solution, (ii) a quantitative approach for measuring the cluster effect on each solver and (iii) a comparison between the branch-and-bound and the SDP approaches. We perform the numerical experiments on a set of 212 challenging problems provided by Kurt Anstreicher. Our findings indicate that SDP relaxations and branch-and-bound have orthogonal difficulties, thus pointing to a possible benefit of a combined method. The following solvers were selected for the experiments: *Antigone 1.1*, *Baron 16.12.7*, *Lindo Global 10.0*, *Couenne 0.5* and *SCIP 3.2*.

## Introduction

Quadratically constrained quadratic programming (QCQP) is the task of finding the global minimum of a linear or quadratic function in a domain defined by finitely many linear and quadratic equations or inequalities. The problem can be written as

$$\begin{aligned} \begin{aligned} \min&\quad f(x) := \frac{1}{2} x^{T}Q_{0}x + c_{0}^{T}x + d_{0}\\ \mathrm{s.t.~}&\quad q_k(x)\in \mathbf{q}_k,\quad k = 1,\ldots , m.\\ \end{aligned} \end{aligned}$$

In this case, $q_{k}(x) := x^{T}Q_{k}x + c_{k}^{T}x + d_{k}$ with $x, c_{k} \in \mathbb {R}^{n}$, $Q_{k} \in \mathbb {R}^{n \times n}$ are symmetric matrices, $d_{k} \in \mathbb {R}$ and $\mathbf{q}_{k}$ are bounded or unbounded closed intervals for $k = 0,\ldots , m$.

QCQP attracted the attention of the optimization community due to its importance in science and engineering. The recent paper [25] proposes a taxonomy for QCQP, review the recent advances in the field and presents a large number of QCQP for testing global optimization software.

The efforts of Chris Floudas advanced the field of QCQPs (and more general global optimization problems) in different directions. We dedicate this paper to his memory. Section 2.1 highlights the software developed by Chris Floudas to cope with QCQPs.

The two trust region subproblem (TTRS) is one of the simplest difficult classes of QCQP. It is defined by a nonconvex quadratic objective and two convex ellipsoidal constraints. Formally, we have

1 $$\begin{aligned}&\min \quad f(x) := \frac{1}{2} x^{T}Qx + c^{T}x\nonumber \\&\mathrm{s.t.~}\quad \Vert Ax - b\Vert \le d_{1},\quad \Vert x\Vert \le d_{2} \end{aligned}$$

where $Q \in \mathbb {R}^{n \times n}$ is symmetric, $c \in \mathbb {R}^{n}$, $A \in \mathbb {R}^{m \times n}$ with $m \le n$ and $d_{1}, d_{2} \ge 0$. We denote by $x^{T}$ the transpose operator and $\Vert .\Vert$ is the Euclidean norm. The TTRS was originally proposed by Celis et al. [15] and hence the problem is sometimes called CDT.

The TTRS received considerable attention from the optimization community for its theoretical and computational aspects. From the theoretical point of view, the problem is interesting since the Hessian of the Lagrangian at the global solution may be indefinite. From the computational perspective, there are algorithms to solve a relaxation of the problem in polynomial time. It is also a well-known result that the Lagrangian dual of the TTRS is a semidefinite program(SDP). Several authors proposed relaxations to the canonical SDP to find out efficient algorithms for solving the TTRS within a specified tolerance. Section 2.2 reviews the recent advances in the TTRS. Section 2.3 describes a set of 212 challenging TTRS instances provided by Anstreicher [6] and used in his paper.

The TTRS was apparently never before studied from the point of view of branch-and-bound (B&B) methods. This paper therefore addresses the following questions

1. Are state-of-the-art B&B solvers capable of solving moderately-sized TTRS within a specified tolerance reliably?
2. How the state-of-the-art B&B solvers are affected by the cluster effect?
3. What is the best option for solving the TTRS within a specified tolerance? Should one rely on SDP-relaxations or B&B methods? Could one benefit from a combined approach?

The first two questions are of interest as a challenge to complete global optimization solvers and to point out possible directions of improvements for them. The third question is important to find out whether B&B methods are a practical alternative to SDP relaxations.

We present our computational study taking all solvers marked as deterministic global on the *GAMS 24.8.2* system into account. In particular, we selected the following solvers: *Antigone 1.1* [33], *Baron 16.12.7* [40], *Couenne 0.5* [9], *Lindo Global 10.0.2539.131* [29] and *SCIP 3.2* [43]. Section 2.4 briefly describes the solvers.

Section 3 addresses the questions 1–3. Section 3.1 details the experimental settings. Section 3.2 presents the reliability analysis. We compare the ability of each solver to complete the search for a solution of (1) under different relative tolerances. The experiment shows that TTRS can be challenging even for relatively small instances for solvers that are not specialized in QCQP. It also points out that the quality of the solution (i.e., the ability to correctly find and recognize the global minimizer) degrades as one decreases the termination tolerance.

Section 3.3 presents a new way to document the cluster effect of a solver and measure this phenomenon in this particular class of problems. We show that the effect is mild on solvers like *Antigone* and *Lindo Global*. On the other hand, *Baron* suffers from the cluster effect in a significant number of problems. The conclusion is not clear for *Couenne* and *SCIP* as they do not finish the search in a large number of instances.

Section 3.4 compares the branch-and-bound and the SDP approaches. We consider the Kronecker second-order cones and the Yang–Burer inequalities proposed in [6] and [46] respectively and compare them with *Baron*. Our findings indicate orthogonal difficulties between the SDP and B&B approaches. Thus, a combined approach for the TTRS and its generalizations appears to be beneficial. We draw some conclusions in Sect. 4.

## Known results

### Contributions by Chris Floudas to QCQP

Many papers by Chris Floudas and his collaborators were concerned with algorithms for global optimization problems that had a direct impact on the solvability of QCQP.

Floudas and Visweswaran presented the Global Optimization Algorithm (*GOP*) [24, 44] which uses the primal-relaxed dual global decomposition. The authors discuss the application of the GOP to quadratic problems in their paper [45].

An important milestone in branch-and-bound methods for global optimization set by the research group of Chris Floudas was the $\alpha$-branch and bound ($\alpha$-*BB*) method [1, 2, 4]. Floudas describes the theory, computational aspects and applications of the $\alpha$-branch and bound method in [23]. Birgin, Floudas and Martinez present a *Fortran* implementation of the method in [11].

In the Global Mixed-Integer Quadratic Optimizer *GloMIQO* [32], Floudas and Misener developed the branch and bound framework so that the solution of QCQPs benefits from dynamically generated cutting planes [34] and piecewise linear and edge concave relaxations [31].

GloMIQO finally evolved into the solver *Antigone* (Algorithms for coNTinuous and Integer Global Optimization of Nonlinear Equations) by Misener and Floudas [33], designed for general mixed-integer global optimization.

### Two trust region subproblems

The TTRS generalizes the trust-region subproblem (TRS). In this particular case, one is interested in the minimization of a quadratic function over a single ellipsoid. The spectral factorization of the Hessian, e.g., as in the *dgqt* routine from *MINPACK-2* [7] is an efficient way to solve the problem. The TRS is the fundamental step for trust region algorithms in nonlinear programming. For a comprehensive approach to trust region methods, see [18]. Yuan [49] discusses the recent improvements in this area.

Yuan [48] proved that the Hessian of the Lagrangian at the global solution of (1) is not necessarily positive semidefinite. The paper also shows that if the Lagrange multipliers are unique at the optimum, then the Hessian of the Lagrangian has at most one negative eigenvalue.

Gaidi [27] studied KKT points of the problem to show that if the Lagrange multipliers are not unique at the global solution of (1), then there exist KKT points where the Hessian of the Lagrangian is positive semidefinite. Peng and Yuan [38] consider optimality conditions for the quadratic problem with two general quadratic constraints.

The difference between the solution of (1) and its Lagrangian dual is called the duality gap. The Lagrangian dual of (1) is a semidefinite program and hence is convex and can be solved efficiently [5]; unfortunately, the duality gap may be significant. Wenbao and Zhang [3] present necessary and sufficient conditions to guarantee that a problem of form (1) admits no duality gap.

Burer and Anstreicher [14] provide a relaxation based on second-order-cone constraints to strengthen the natural SDP relaxation, thereby reducing the duality gap in many cases. Beck and Eldar [8] propose a different approach based on a quadratic mapping between the image of the real case and a definition of the problem over the complex plane. Bomze and Overton [12] study the gap by the copositivity perspective. Yuan et al. [47] show that one can narrow the duality gap by adding an appropriate second-order-cone constraint to (1). Yang and Burer [46] study the case $n = 2$ in detail to fully characterize the second-order-cone constraints and show that their results are also useful in higher dimensions.

Bienstock [10] presents a polynomial-time algorithm for quadratic programming with a fixed number of quadratic constraints. His algorithm relies on a relaxed definition of feasibility and, for a given $\epsilon \in {]0, 1[}$, either (i) provides a certificate that (1) is infeasible, or (ii) computes a point $x^{*}$ such that $\Vert Ax^{*} - b\Vert \le d_{1}+\epsilon$, $\Vert x^{*}\Vert \le d_{2}+ \epsilon$, and the global minimum $\hat{x}$ satisfies $f(\hat{x}) \le f(x^{*})+ \epsilon$. The Bienstock algorithm seems to be more of theoretical than practical interest. Bienstock’s algorithm seems to be exponential in $\varepsilon ^{-1}$, which is not promising. Indeed, to the best of our knowledge, it has never been implemented with reported results.

Takeda et al. [39] propose a different polynomial-time algorithm that relies on the computation of all Lagrange multipliers of (1) via the two-parameter linear eigenvalue problem. The paper presents numerical experiments in a *C++* implementation of the method that is not publicly available.

Further work on TTRS can be found in [16, 17, 22, 28, 30, 37, 50]. These references were included in the text for completeness. In the present context, they are marginal, and in our opinion their discussion does not merit the additional space.

### The test problem collection

Burer and Anstreicher [14] generated a set of 1791 instances of form (1) with $n \in \{5, 10, 20\}$ for the purpose of testing the strength of semidefinite programming (SDP) relaxations. Most of these problems were solved to $\varepsilon$-optimality ($\epsilon = 10^{-4}$) by the standard SDP relaxation. The other—more difficult—212 instances were sent to us by Kurt Anstreicher in the fall of 2016 and constitute our test set. It consists of 38 problems of size 5, 70 problems of size 10 and 104 problems of size 20. The test set is available in the *GAMS*, *AMPL* and *COCONUT* formats at http://www.mat.univie.ac.at/~montanhe/publications/ttrs.zip.

The supplementary file also contains the results of our numerical experiments and scripts to reproduce the analysis in this paper.

In a recent paper, Anstreicher [6] presents new constraints for the SDP relaxation of (1). In particular, he performs numerical experiments with his new method and the one discussed in [46]. Both approaches combined can solve 156 of the set of 212 unsolved instances from [14] within a tolerance of $\epsilon = 10^{-4}$. In our experiments, we classify the set 156 instances as easy. The remaining 56 instances are classified as hard.

**Fig. 1:**
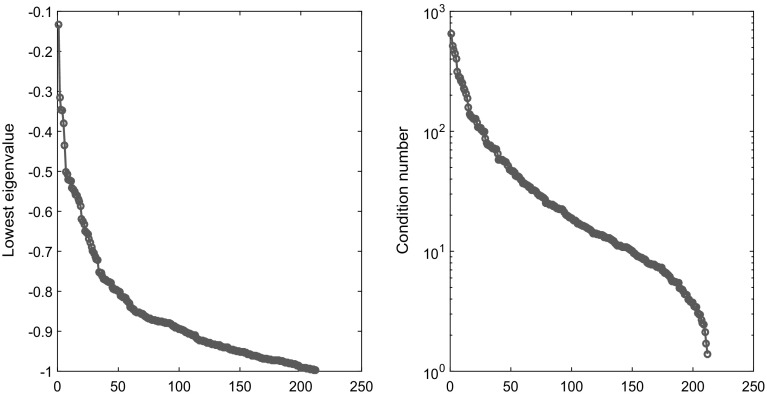
(Left) Sorted lowest eigenvalues of the matrices *Q* in the test collection. (Right) Sorted condition numbers of the matrices *Q* in the test collection

For all 212 instances, the matrix *A* is diagonal with positive entries, *b* is the zero vector and *Q* is dense with no zero entries. Figure 1 displays the sorted lowest eigenvalues and condition number of the matrices *Q* in the test collection.

### Complete global optimization solvers

Complete solvers can find one or all global minimizers within an $\epsilon$ tolerance, assuming exact computations. This is guaranteed by using a branch and bound framework in which the bounds are obtained by (in exact arithmetic) mathematically correct estimation procedures. For our study we used five different complete solvers.

*Antigone* was already introduced in Sect. 2.1.

*Baron* [40] stands for Branch And Reduce Optimization Navigator. It implements a spatial branch and bound algorithm that computes lower bounds for each subproblem utilizing linear relaxations and duality theory. It also relies on bound tightening techniques such as probing and violation transfer.

*Couenne* [9] is the Convex Over-and Under-ENvelopes for Nonlinear Estimation. It is an open source branch and bound algorithm that, similarly to BARON, obtains a lower bound through an LP relaxation using the reformulation techniques. It also implements several bound tightening procedures as well as a recently introduced feasibility pump heuristic, a separator of disjunctive cuts, and different branching schemes including strong, pseudo-cost, and reliability branching. Source code and documentation are available online at https://projects.coin-or.org/Couenne.

*SCIP* [43] (derived from Solving Constrained Integer Problems) started as a MILP solver but evolved into a nonconvex MINLP solver. It follows the same approach of BARON and Couenne, implementing a branch and bound algorithm with linear relaxation and bound-tightening procedures. Source code and documentation are available at the project website http://scip.zib.de.

*Lindo Global* [29] finds global optima to nonconvex, nonlinear and integer mathematical models using a branch and bound/relax approach. It allows a wide range of mathematical functions, including nonsmooth, trigonometric, logical and statistical. While earlier versions (such as the one tested in [36]) were based on mathematical bounding procedures, the results reported below suggest that the most recent version tested contains additional heuristics that sacrifices any guarantee of global optimality.

## Numerical results

### Experimental setup

We ran the 212 instances from the test set with each of the complete global solvers *Antigone 1.1*, *Baron 16.12.7*, *Couenne 0.5*, *Lindo Global 10.0.2539.131*, and *SCIP 3.2*. We ran the experiments on four identical machines, each with 8 GB of RAM memory and a core i5-4670 processor with 800 MHz of processor capacity on each core. The operating system on each machine was Ubuntu 14.05. For each test case, we set a relative optimality tolerance of $\epsilon \in \{10^{-8},10^{-7},\ldots ,10^{-4}\}$.

**Table 1:** Reliability analysis for the complete global optimization solvers with different termination tolerances

Solver	$\epsilon = 10^{-4}$			$\epsilon = 10^{-5}$			$\epsilon = 10^{-6}$		
	G+ (%)	G!/G+ (%)	WC (%)	G+ (%)	G!/G+ (%)	WC (%)	G+ (%)	G!/G+ (%)	WC (%)
Antigone	99	87	0	99	83	0	98	80	0
Baron	100	98	0	100	97	0	99	97	0
Couenne	100	50	0	100	50	0	99	51	0
LindoGlobal	90	100	9	83	100	16	78	100	21
SCIP	88	51	0	82	55	0	82	56	0

Column $G{+}$ displays the percentage of globally solved problems, where a global solution was found within the specified tolerance. The ratio $\frac{G!}{G{+}}$ stands for the percentage among globally solved problem where a global optimum was claimed. Column *WC* presents the percentage of wrong claims

**Table 2:** Reliability analysis continued

Solver	$\epsilon = 10^{-7}$			$\epsilon = 10^{-8}$		
	G+ (%)	G!/G+ (%)	WC (%)	G+ (%)	G!/G+ (%)	WC (%)
Antigone	98	80	0	76	85	38
Baron	99	96	0	78	89	39
Couenne	99	51	0	8	23	74
LindoGlobal	78	100	21	62	100	55
SCIP	79	54	2	68	65	28

**Fig. 2:**
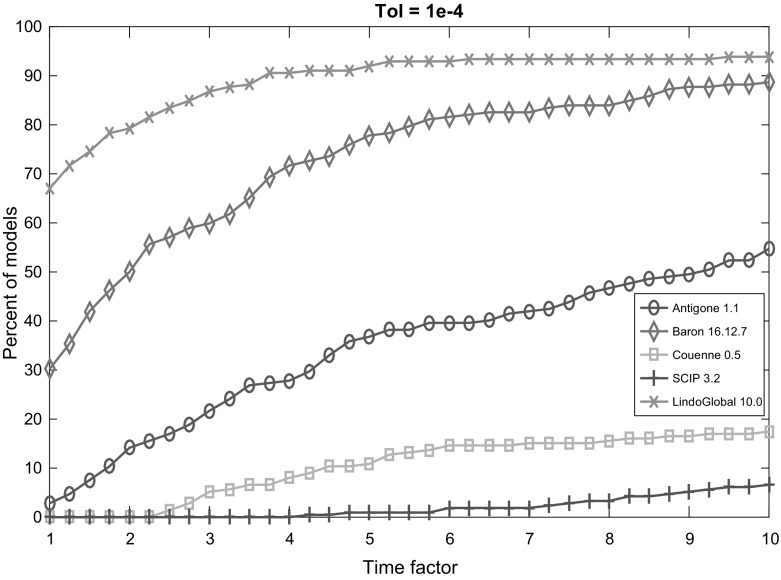
Performance profile for each solver with termination tolerance $10^{-4}$

**Fig. 3:**
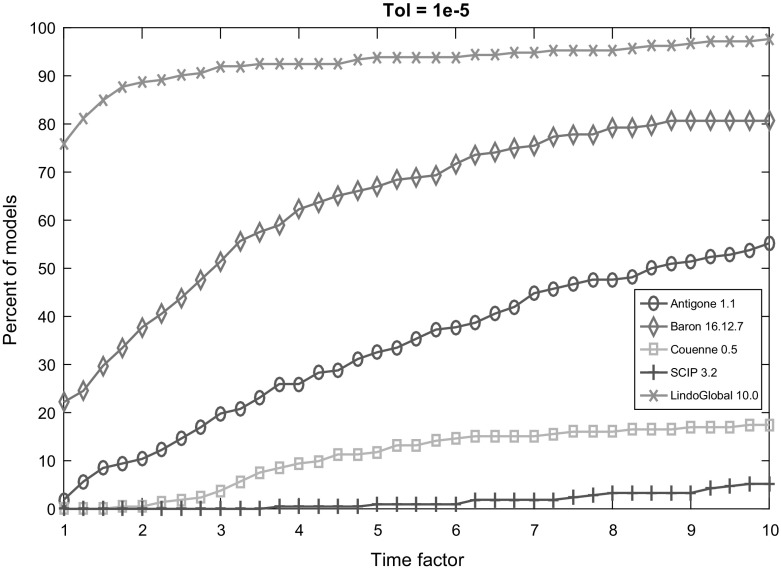
Performance profile for each solver with termination tolerance $10^{-5}$

**Fig. 4:**
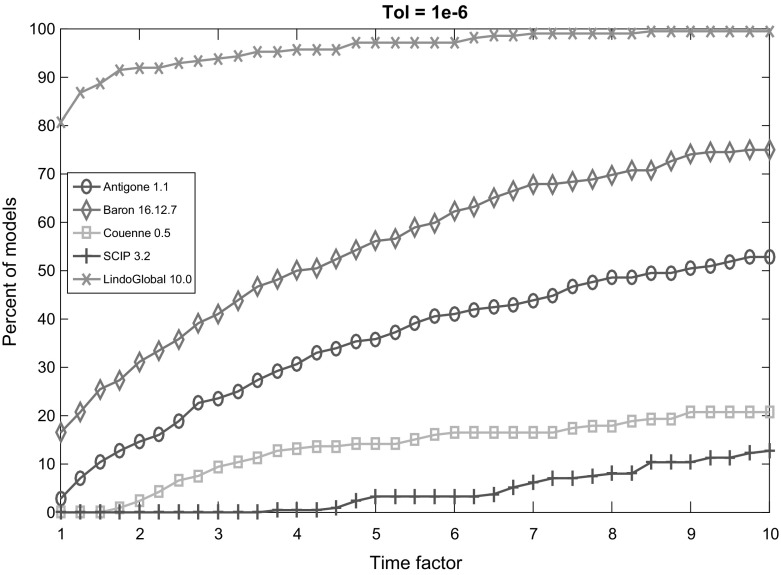
Performance profile for each solver with termination tolerance $10^{-6}$

**Fig. 5:**
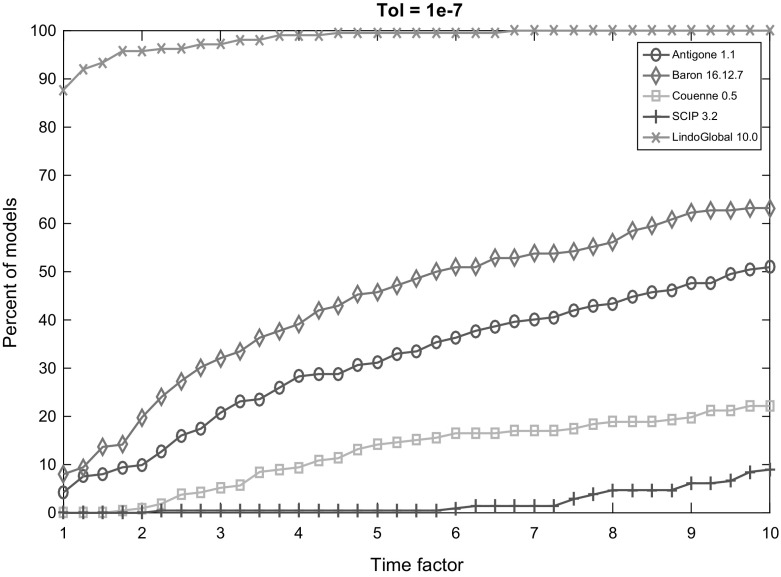
Performance profile for each solver with termination tolerance $10^{-7}$

**Fig. 6:**
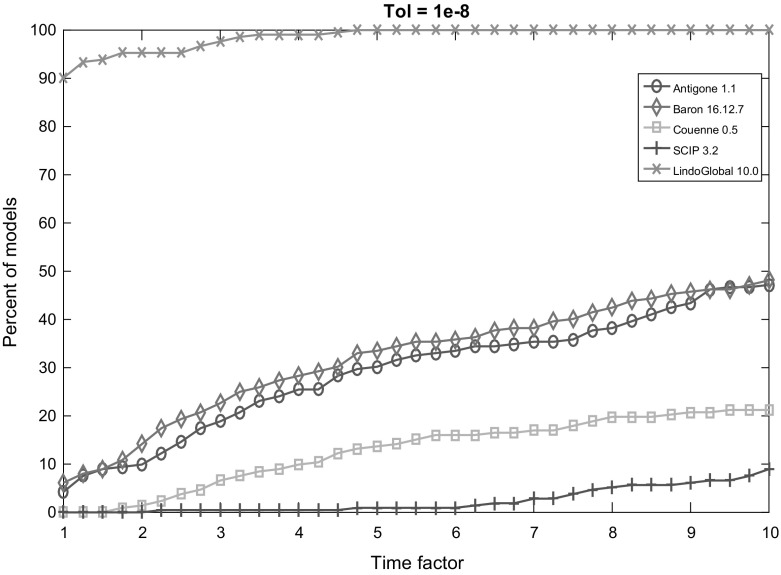
Performance profile for each solver with termination tolerance $10^{-8}$

**Fig. 7:**
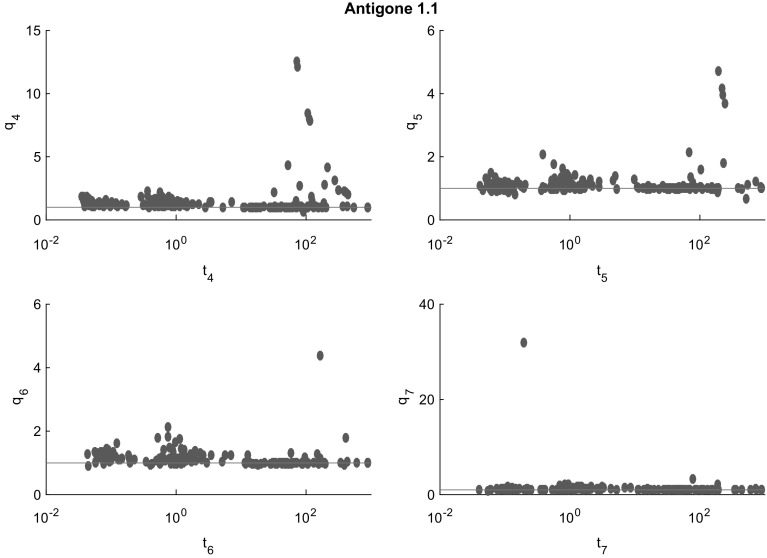
Cluster effect analysis for Antigone. With relatively few exceptions, the cluster effect is suppressed or mild. This may be due to special techniques implemented only in Antigone for quadratic problems

**Fig. 8:**
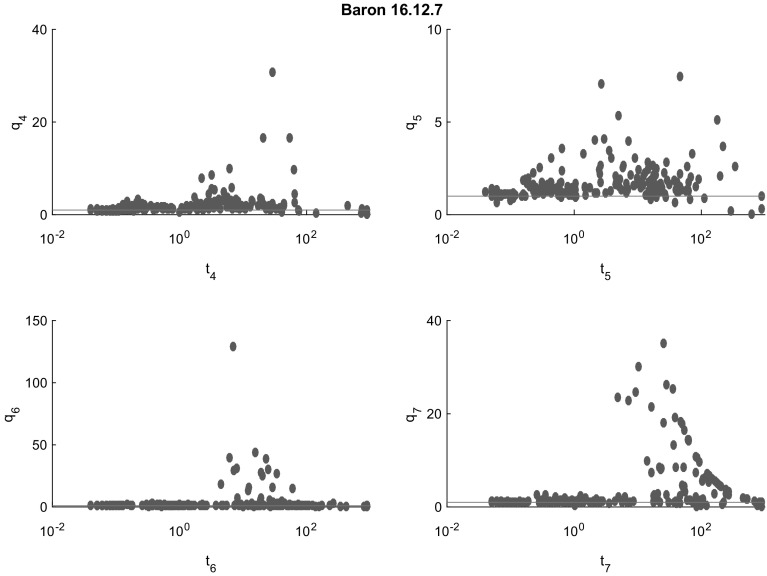
Cluster effect analysis for Baron. There are a significant number of problems with a severe cluster effect, except for the easily solved problems

**Fig. 9:**
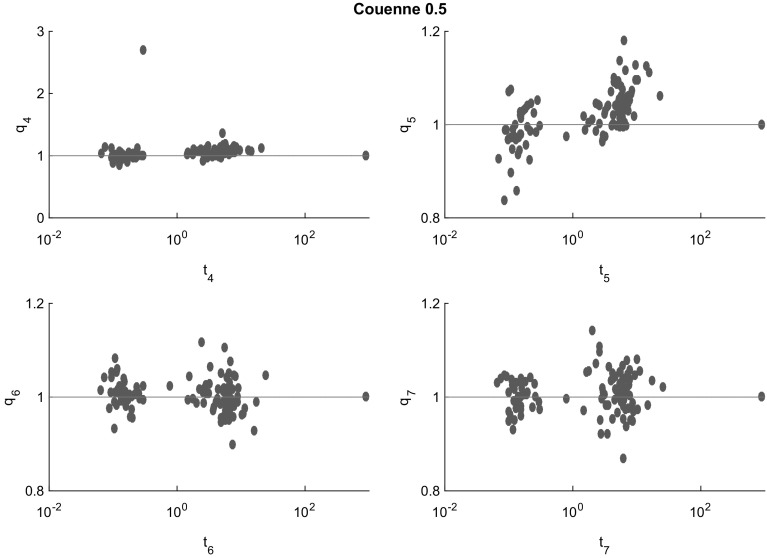
Cluster effect analysis for Couenne. In the problems solved to completion, the cluster effect is virtually absent. But this is mainly due to the fact that Couenne fails to complete the search in a large proportion of cases, and this failure is due to a cluster effect not visible in the present analysis

**Fig. 10:**
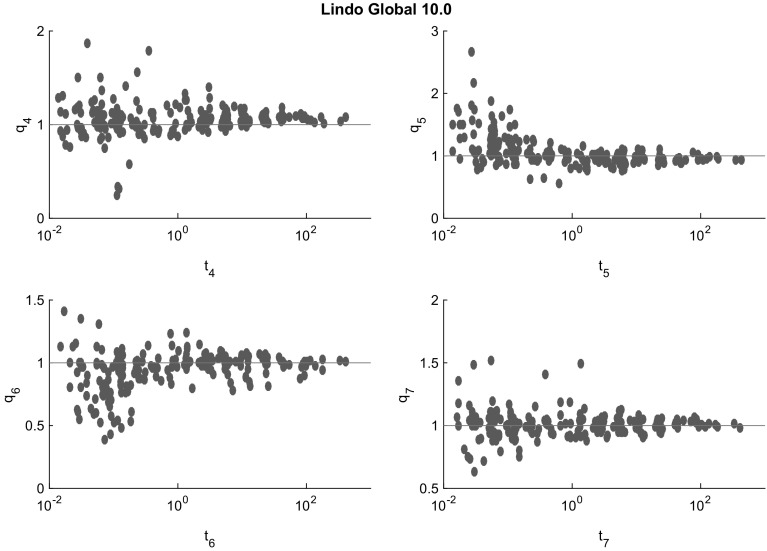
Cluster effect analysis for LindoGlobal. There is virtually no visible cluster effect. This may be related to heuristics that also leads to the lack of reliability of LindoGlobal

**Fig. 11:**
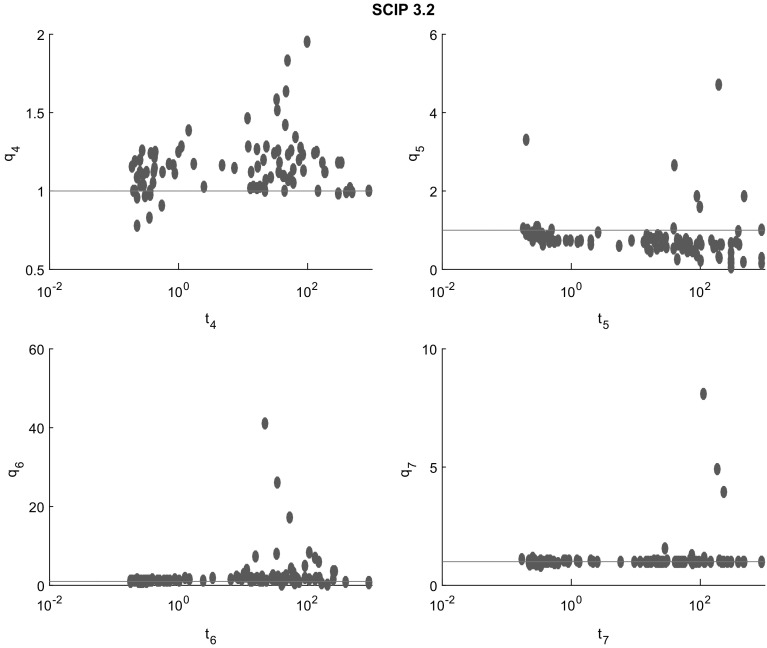
Cluster effect analysis for SCIP. With relatively few exceptions, the cluster effect is suppressed or mild. But due to the low efficiency of SCIP, this conclusion is much less reliable than for Antigone

We performed the experiments on the *GAMS 24.8.2* system [13] with the options ${\text {optca}} = 0$, ${\text {optcr}} = \epsilon$, ${\text {decimal}} = 8$, and ${\text {m.workspace}} = 32$. We imposed a time limit of $15~\hbox {min} = 900~\hbox {s}$. However, in a number of cases, a solver did not respect the time limit imposed. In such cases, the job was killed automatically after 930 s. In case of a timeout, we set the time to 900 s.

### Reliability analysis

This subsection addresses the question: Are state-of-the-art B&B solvers capable of solving moderately-sized TTRS within a specified tolerance reliably?

To answer the question, we consider the experimental setup discussed in Sect. 3.1 and analyzed the results using the publicly available Optimization Test Environment [19]. The software is designed to automatically generate the reliability analysis from the output of any local or global solver. The program solCheck from the test environment verifies the accuracy of an approximate solution point. The maximal constraint violation is required to be at most solCheckTolerance, and the maximal deviation of the objective function value from the optimal value (the best value known to us) is required to be at most maxGlobalError. More precisely, the solution $x^{*}$ found by a solver is accepted as a valid global minimum whenever it satisfies

$$\begin{aligned} f(x^{*}) - f\big (x^{{ best}}\big ) \le {\text {maxGlobalError}} * d \end{aligned}$$

where $x^{{ best}}$ is the best known solution for the problem and $d = 1$ if

$$\begin{aligned} h := \min \big (f(x^{*}),f\big (x^{{ best}}\big )\big ) \le 1, \end{aligned}$$

and $$d = h$$ otherwise.

Given a solver tolerance of $\epsilon =10^{-i}$, the solCheckTolerance (for the constraints violation analysis) and the maxGlobalError (for the objective function analysis) used were $10^{-(i-1)}$ for $i = 4,5,6$ and $10^{-(i-2)}$ for $i = 7,8$. We set a problem as solved by a solver if solCheck verifies the accuracy of the approximate solution returned. In all other cases, we considered the result as a failure, irrespective of the status message of the solver.

However, we recorded if the solver reported that a global optimum had been found, and checked whether this was indeed the case. The solvers Baron, Antigone and Lindo Global provide their own termination messages, which not always agreed with the output given by GAMS. In these cases, we recorded the (more useful) output given by the solver. For SCIP and Couenne we used the termination status provided by GAMS.

Tables 1 and 2 summarize the reliability analysis. Column G+ reports the ratio of globally solved problems. Column G!/G+ gives the percentage among globally solved problems where a global optimum was claimed. In WC, we display the portion of wrong claims, i.e., cases where a global optimum was claimed and no timeout occurred, but solCheck could not verify that the solution was optimal within the specified tolerance.

**Fig. 12:**
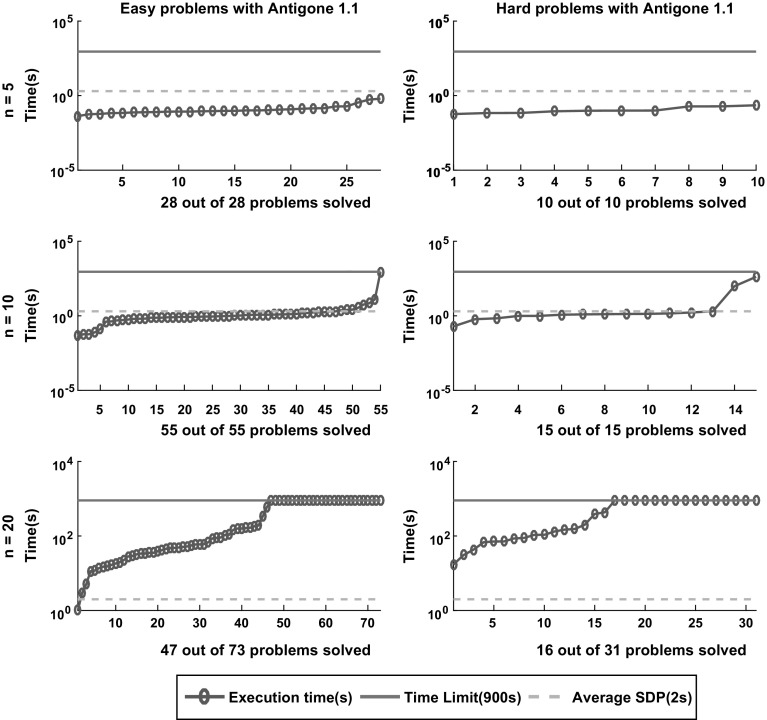
Sorted execution time for *Antigone* to solve the 212 instances classified as easy and hard according to the definition in Sect. 2.3 with termination tolerance of $\epsilon = 10^{-4}$. The dashed lines displays the average time of the SDP relaxation approach reported in [6]

**Fig. 13:**
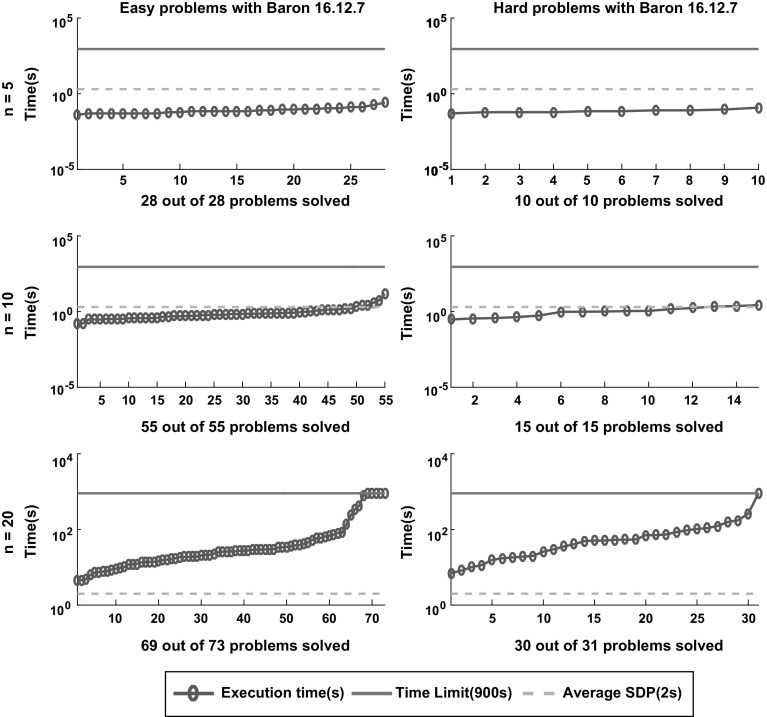
Sorted execution time for *Baron* to solve the 212 instances classified as easy and hard according to the definition in Sect. 2.3 with termination tolerance of $\epsilon = 10^{-4}$. The dashed lines displays the average time of the SDP relaxation approach reported in [6]

**Fig. 14:**
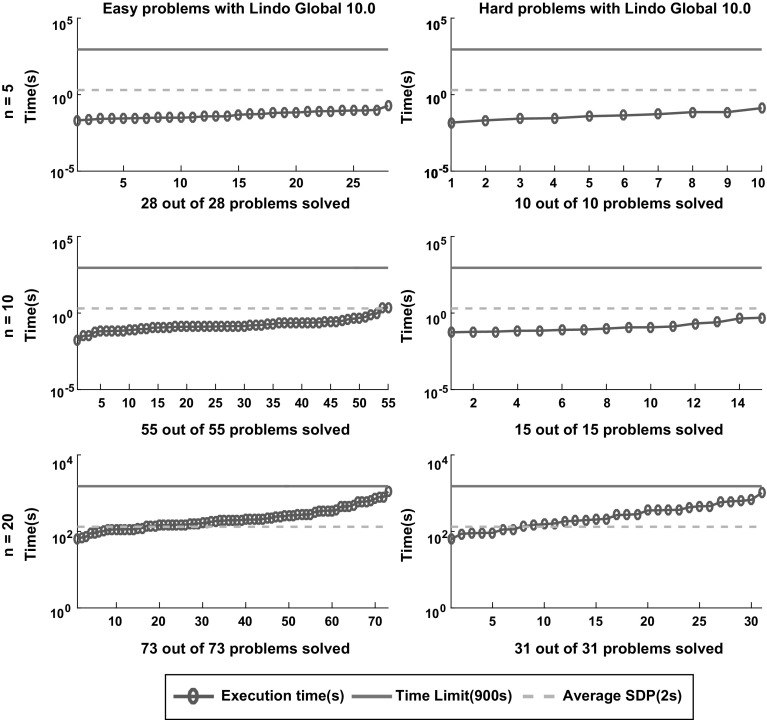
Sorted execution time for *Lindo Global* to solve the 212 instances classified as easy and hard according to the definition in Sect. 2.3 with termination tolerance of $\epsilon = 10^{-4}$. The dashed lines displays the average time of the SDP relaxation approach reported in [6]

**Fig. 15:**
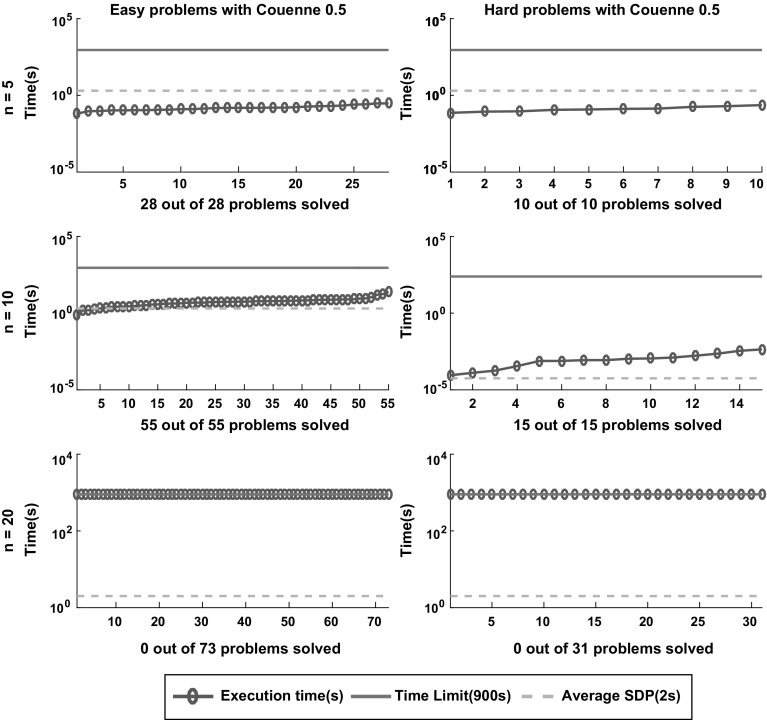
Sorted execution time for *Couenne* to solve the 212 instances classified as easy and hard according to the definition in Sect. 2.3 with termination tolerance of $\epsilon = 10^{-4}$. The dashed lines displays the average time of the SDP relaxation approach reported in [6]

**Fig. 16:**
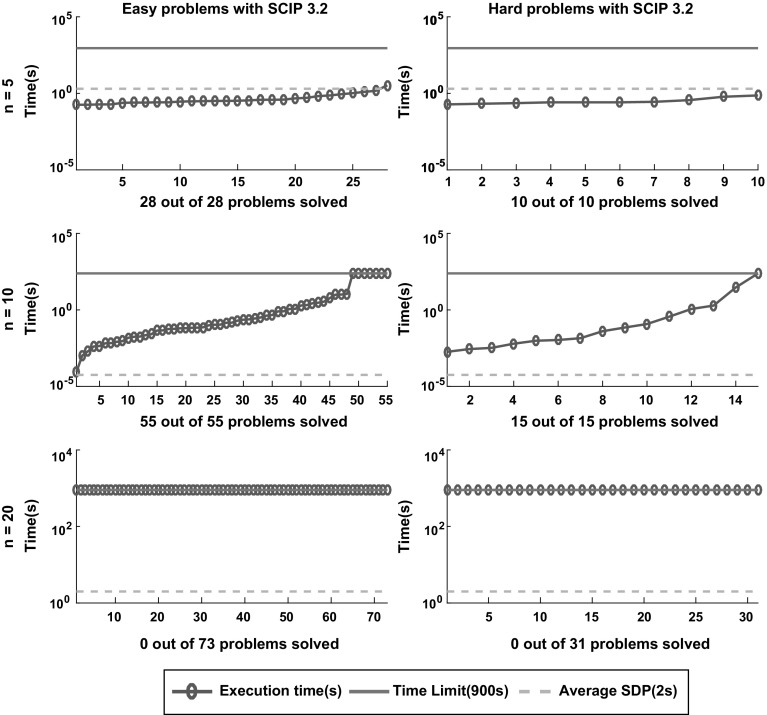
Sorted execution time for *SCIP* to solve the 212 instances classified as easy and hard according to the definition in Sect. 2.3 with termination tolerance of $\epsilon = 10^{-4}$. The dashed lines displays the average time of the SDP relaxation approach reported in [6]

We see from the tables that LindoGlobal reports a high percentage of wrong claims on problems where the other solvers conclude the search correctly. We observed that LindoGlobal does not update the lower bound on the objective function value during the search for any instance. Thus, it seems that it bases its claims not on a successful reduction of the duality gap during the branch-and-bound procedure but based on some far less reliable heuristics. The large percentage of wrong claims for all solvers when $\epsilon =10^{-8}$ (and for SCIP already some false claims for $\epsilon =10^{-7}$ indicates that conditioning issues and the lack of rounding error control in all solvers significantly degrade the reliability when high accuracy is requested.

Figures 2, 3, 4, 5 and 6 (after references) display the time performance profiles for each solver with varying tolerances. The combined analysis of the performance profiles and the Tables 1 and 2 show that *Baron* gives the best balance between the reliability of the solution and the efficiency. *Antigone* is also a good option if one takes the reliability of the solver into account. It is due to the fact that *Antigone* implements several state-of-the-art methods for QCQP. *Couenne* and *SCIP* perform comparatively poorly. Finally, if only the efficiency of the solver is the factor of choice, then *Lindo Global* is the best solver in our experiment.

### Cluster effect

We now consider the question“How the state-of-the-art B&B solvers are affected by the cluster effect?”

Branch and bound methods frequently suffer from the cluster effect. The phenomenon was first described in [21] as the excessive splitting of the search domain close to the global optima. The resulting boxes form a cluster around the solution, and in a branch-and-bound approach, the pattern repeats itself at smaller and smaller scales, leading to a severe slowdown when high accuracy is requested.

Discarding efficiently the boxes in such a cluster is a highly nontrivial challenge since it needs special techniques involving exclusion regions. Exclusion regions are portions of the search domain where one can prove the existence and uniqueness of a local solution, and hence needs no further subdivision. Exclusion regions are typically constructed using a Krawczyk operator for the first order optimality conditions, as described in [35]. Third order methods to build exclusion regions for systems of equations are presented in [42] and the extension for global optimization problems is the subject of [41]. For QCQPs, the third order terms vanish, leading to simplifications in the resulting algorithms. The constraint aggregation method described in [20] also aims at reducing the cluster effect. Exclusion regions in the context of multi-objective optimization are discussed in [26].

None of these techniques are built into current state-of-the-art solvers. The latter try instead to avoid the cluster effect by providing only a $\epsilon$-optimal solution. The program stops when it has shown that there is no feasible point with an objective value of $f^{*}-\epsilon$, where $f^{*}$ is the function value of the best feasible point found so far. While this reduces the severeness of the cluster effect, the branch-and-bound process may discard better points whose function value is in the interval $[f^{*}-\epsilon ,f^{*}]$. In particular, unlike in the approach via exclusion regions, no bounds on the accuracy of the approximate optimizer returned can be obtained.

If $\epsilon$ is not too small, this approach is reasonable and produces satisfactory solutions for many practical applications. However, it becomes inefficient if one needs to guarantee a high accuracy of the optimal value.

Figures 7, 8, 9, 10 and 11 (after references) quantify the cluster effect for each solver on our test set by displaying for each $i=4,5,6,7$ (and each solver) a scatterplot of points corresponding to the problems solved successfully. We denote the time needed to solve the problem at the tolerance $\epsilon = 10^{-i}$ by $t_{i}$ and define the quotient

$$\begin{aligned} q_{i} := \frac{t_{i+1}}{t_{i}}. \end{aligned}$$

In addition, a reference line for $q_i=1$ is drawn. Note the different scales of the vertical axes.

The cluster effect is effectively suppressed if the quotient $q_i$ is close to or below 1. (Values $q_i<1$ mean that the higher accuracy problem was solved faster than the lower accuracy one. This may be due to inaccuracies in the measured cputime, or to the fact that a better feasible point found by a more accurate local search lead to better pruning in later steps.) Values of $q_i$ around 2–5 indicate the presence of a mild cluster effect, while values of 10 or more indicate a severe cluster effect.

Figures 7, 8, 9, 10 and 11 show that several points in the scatterplot fall below the reference line $q_{i} = 1$. It means that the time needed to solve the problem with termination tolerance $\epsilon = 10^{-i-1}$ is lower than the time needed to solve the same instance with $\epsilon = 10^{-i}$. We observe this behavior only in instances where the execution time is significantly smaller than 1 s and therefore it is probably caused by small differences in the choices of each solver during the execution.

### Comparing SDP and branch and bound on TTRS

We looked into the question whether the difficulty of problems concerning the quality of SDP relaxations is related to the difficulty of problems for the current generation of branch-and-bound solvers.

In Yang and Burer [46] and Anstreicher [6], all but $56=10+15+31$ of the 212 test problems were solved with more sophisticated semidefinite relaxations, using SOC-RLT cuts and Kronecker product constraints, respectively. According to [6], the average time to solve an instance with tolerance of $\epsilon = 10^{-4}$ and $n=20$ is 2 s.

Figures 12, 13, 14, 15 and 16 (after references) show the time needed for each branch and bound solver to conclude the task with $\epsilon =10^{-4}$ considering both categories of problems, easy and hard. We also display the average time described in [6] and the time limit established in our experiment.

One can easily see that most solvers can solve problems with $n = 5$ and $n = 10$ efficiently and independent from the hardness. In particular, the average time for each B&B solver to finish the search in the case $n = 5$ is lower than the average time required by the SDP relaxations. For $n = 10$ one can see that *SCIP* is the only one with average time significantly greater than the SDP approach. Finally, *Couenne* and *SCIP* could not solve any instance with $n = 20$ within the time limit of 900 s, while *Baron* and *Antigone* were competitive in time with the SDP approach. Regarding the correctness of the solution, note from Table 1 that for $\epsilon = 10^{-4}$, *Lindo Global* is the only one with 9% of wrong claims while *Baron* and *Antigone* can find and recognize a global solution with probabilities 98 and 87% respectively. We also note that every instance not solved by *Baron* can be solved by SDP-relaxations.

In a branch-and-bound method, semidefinite relaxations can in principle be applied at the root node, solving the problem in the ”easy” cases. In the remaining, ”hard” cases, a branch-and-bound method appears to be effective but somewhat slow. Solving an SDP relaxation at selected nodes could reduce the number of nodes needed and hence accelerate the convergence rate of the overall process. On the other hand, solving one of the powerful SDP relaxations consumes significantly more time than the time spent otherwise on a node. Thus while there is potential for combining the approaches, it needs good judgment in how the combination is done. We are not aware of any complete solver which combines SDP relaxations and branch-and-bound.

## Conclusions

In conclusion, we may state that for moderate tolerances ($\epsilon \ge 10^{-7}$), Baron is the best and most reliable global solver among the 5 solvers tested. Antigone is second best if reliability matters, whereas LindoGlobal is second best if one disregards claims of global optimality. Couenne and SCIP perform comparatively poorly and need a considerable strengthening to become competitive with the other solvers. All solvers become unreliable when a high accuracy such as $\epsilon =10^{-8}$ is requested.

Our analysis of the cluster effect indicates that Baron could benefit from an improved treatment of the end game in the branch-and-bound process, where the cluster effect dominates the time needed to handle the boxes very close to the solution.

We also observed that SDP relaxations and branch-and-bound have orthogonal difficulties. This points to a possible benefit for a combination approach.
